# Oxymatrine protects against l-arginine-induced acute pancreatitis and intestine injury involving Th1/Th17 cytokines and MAPK/NF-κB signalling

**DOI:** 10.1080/13880209.2019.1657906

**Published:** 2019-09-08

**Authors:** Zhiqiang Zhang, Qingfeng Liu, Hui Zang, Qingliang Shao, Tian Sun

**Affiliations:** a Department of General Surgery, The People's Hospital of Liaoning Province, Shenyang, China;; b Department of General Surgery, The People's Hospital of China Medical University, Shenyang, China

**Keywords:** Intestinal barrier, inflammation, IL17, ROR-γt, T-bet, ERK, P38

## Abstract

**Context:** Oxymatrine (OMT) has various pharmacological effects, including immune reaction regulation, anti-inflammation and anti-hypersensitive reaction.

**Objective:** This is the first report to investigate the molecular mechanism of OMT function in l-arginine (Arg)-induced acute pancreatitis (AP) involving intestinal injury.

**Materials and methods:** Rat pancreatic AR42J and small intestinal IEC-6 cells were treated with Arg (200–800 µM) for 48 h plus OMT (4 mg/mL) treatment. Thirty adult Wistar rats were randomly assigned to control (saline), AP (i.p. of 250 mg/100 g body weight Arg) and OMT (i.p. injection of 50 mg/kg b.w. OMT every 6 h following Arg). Both cells and rats were harvested at 48 h.

**Results:** Arg-induced cell proliferation in both rats AR42J (EC_50_ 633.9 ± 31.4 µM) and IEC-6 cells (EC_50_ 571.3 ± 40.4 µM) in a dose-dependent manner, which was significantly inhibited by OMT (4 mg/mL). Meanwhile, Arg (600 µM) induced expression of proinflammatory cytokines (TNF-α, IL-6, IL-1β, NF-κB, IL-17A/IL-17F and IFN-γ) and activation of p-p38/p-ERK *in vitro*, which was reversed by OMT. *In vivo*, OMT (50 mg/kg) inhibited 250 mg/100 g of Arg-induced AP involving intestinal injury, including inhibiting Arg-induced inflammatory in pancreas and intestine, inhibiting Arg-induced increase of TNF-α, IL-6, IL-1β, NF-κB and p-p38/p-ERK-MAPK signalling, and inhibiting Arg-induced increase of IL-17A/IL-17F, IFN-γ, ROR-γt and T-bet. Meanwhile, OMT inhibited Arg-induced expression of CD44 and CD55 in intestinal injury.

**Discussion and conclusions:** OMT protects against Arg-induced AP involving intestinal injury via regulating Th1/Th17 cytokines and MAPK/NF-κB signalling, which is a promising therapeutic agent in clinics.

## Introduction

Acute pancreatitis (AP) is not only a local pancreatic inflammation but also a systemic disease involving multiple organs. The intestinal barrier dysfunction plays a pivotal role in AP progression (Chen et al. 2017c). AP involving intestinal barrier injury was associated with excessive release of inflammatory cytokines, damage in intestinal epithelium and bacterial translocation (Zhang et al. 2007). Though the close interaction of AP with intestinal injury is reported previously (Wang et al. 2009; Xu et al. 2014), the effective intervention and potential molecular mechanism have not been fully elucidated.

**Figure 1.**
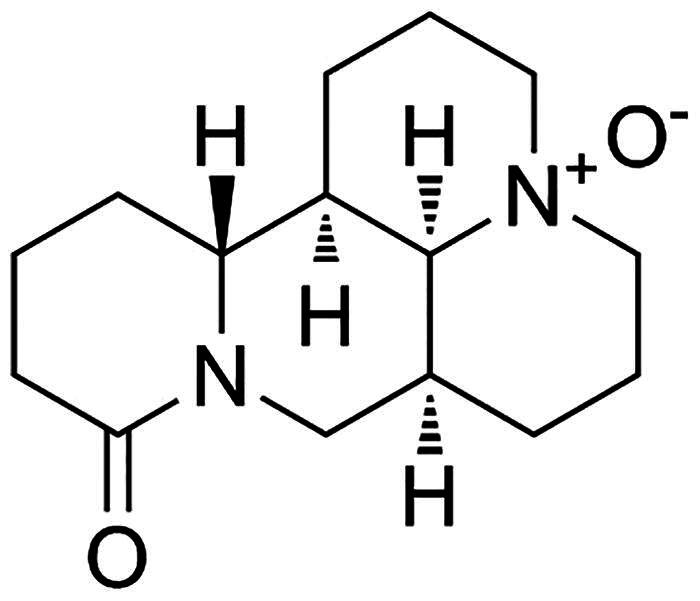
OMT (matrine oxide, matrine *N*-oxide, matrine 1-oxide) is one of many quinolizidine alkaloid compounds extracted from the roots of *Sophora flavescens*, a Chinese herb.

Arginine (Arg), a semi-essential amino acid in protein synthesis, plays a significant role in regulating immune response, hormone secretion and wound healing (Zeng et al. 2018). Arg-induced AP model has been successfully constructed in previous studies (Chen et al. 2017a). Oxymatrine (OMT), one of quinolizidine alkaloid compounds extracted from the root of *Sophora flavescens*, a Chinese herb (Figure 1), plays a critical role in regulating hypersensitive and immune reaction, histamine release and inflammation (Chen et al. 2001). Recently, numerous studies have focused on its function in inflammation and the balance between T (Treg) and T helper cells (Th) *in vitro* and *in vivo*. For example, OMT exhibits a protective role in rheumatoid arthritis (RA) through mediating inflammation and Treg/Th17 in CIA rats (Ma et al. 2017). OMT ameliorates ulcerative colitis (UC) through inhibiting inflammation and the differentiation of Th1 and Th17 cells (Chen et al. 2017b). Our previous study showed Arg-induced AP was reversed by OMT (Zhang et al. 2012). However, the molecular mechanism of OMT in Arg-induced AP and subsequent intestinal injury, to our knowledge, hasn’t been reported.

## Materials and methods

### Animals

Forty male adult Wistar rats, weighting 250–300 g, were supplied from Beijing Vital River Laboratory Animal Technology (Beijing, China). The animals were kept from the specific pathogen-free (SPF) Animal Experimental Ministry of China Medical University. All animals were housed in plastic cages containing wood shaving and maintained in a room at 25 °C with a 12 h light/night cycle with free access to standard laboratory diet and water. Animals were maintained according to institutional regulations in facilities approved by the Animal Care Committee of China Medical University in accordance with Chinese government guidelines for animal experiments.

### Experimental protocol

For detecting OMT effect in normal rats, 10 rats were randomly assigned to two groups (*n* = 5): control (saline treatment) and OMT (OMT treatment). The dose of Arg and OMT follows our previous study (Zhang et al. 2012). OMT group: AP rats received i.p. injection of 50 mg/kg OMT (Abcam, Cambridge, UK) every 6 h, a total four times in 24 h. The control group received an equal volume of 0.15 M physiological saline under the same condition.

For detecting OMT effect in Arg-treated rats, 30 rats were randomly assigned to three groups (*n* = 10): control (saline treatment), AP (Arg treatment) and OMT (OMT treatment after Arg induction). As described previously (Takács et al. 2002; Zhang et al. 2012), the rats of AP group were induced by i.p. injection of 250 mg/100 g b.w. of Arg (Sigma, St. Louis, MO, USA) twice (at an interval of 1 h). The control group received an equal volume of 0.15 M physiological saline under the same condition. OMT group: AP rats that were induced by the administration of Arg received an i.p. injection of 50 mg/kg OMT (Abcam, Cambridge, UK) every 6 h, a total four times. All rats were sacrificed at 48 h. The pancreas and terminal ileum were removed for later HE, IHC, PCR and WB assays.

### Cell culture and treatment

The non-transformed rat pancreatic AR42J cells and rat small intestinal IEC-6 cells (Bena Culture Collection, Beijing, China) were cultured as previously described (Liu et al. 2009). For detecting OMT effect in normal rat cells, AR42J and IEC-6 cells plated in 6-well plates were treated with OMT (4 mg/mL) and same volume of saline (control) for 24 h as described previously (Zhang et al. 2012; Guzman et al. 2013).

For detecting OMT effect in Arg-induced rat cells, AR42J and IEC-6 cells were treated with Arg (600 µM) for 48 h with or without OMT (4 mg/mL) treatment for the last 24 h. The optimum concentration of Arg (600 µM) and OMT (4 mg/mL) was calculated from our pre-experiment (MTT assays shown in the ‘Results’ section). Cells were starved at 2 h with serum-free media prior to incubation with Arg or OMT.

## MTT assays

AR42J and IEC-6 cells growth under the various doses of Arg combing with or without OMT (4 mg/mL) was detected by MTT. Briefly, cells were harvested, counted and then seeded into 96-well plates at the density of 8000 viable cells per well overnight. After starvation (2 h), Arg was added to the media with various concentrations shown in the ‘Results’ section. Cells were incubated with 10 µL of MTT (5 mg/mL in PBS, Sigma) for 4 h at 37 °C. The media was then removed and 150 µL of dimethyl sulphoxide (Sigma) was added to each well. Each experimental plate was read on ELISA 96-well microtiter plate reader (BIORAD680, USA) at 570nm wavelength. Experiments were performed in triplicates, and data were presented as the percentage of treated cells compared with control cells. We using half maximal effective concentration EC_50_ to calculate the drug function in Arg and Arg plus OMT-treated groups, respectively.

### Morphological examination

Terminal ileum specimens (5 cm) were stained with haematoxylin and eosin (HE) and blindly examined under microscopy by three special pathologists. As described previously (Howarth et al. 1996), a total score was derived from the sum of 11 histologic criteria, including villus fusion and stunting, damage of the brush border, the number of goblet cells, crypt loss, architectural disruption, injury of crypt cells, crypt abscess formation, polymorphonuclear cells and lymphocytes infiltration, lymphatics and capillaries dilatation, and submucosal and muscularis external layers edema. Each histologic variable was scored from 0 (normal) to 3 (maximal damage) to consider a maximum possible 33 scores for each intestinal sample.

### Real-time PCR

Total RNA was extracted from AR42J and IEC-6 cells and intestinal tissue samples from control, AP and OMT groups with TRIZOL reagent under the manufacturer (Takara Bio, Otsu, Japan). The expression of target genes was analyzed in a light cycler 2.0 with the light cycler kit (Takara). The conditions were as follows: 95 °C for 30 s, 40 cycles of 95 °C for 5 s and 60 °C for 30 s. The primers are summarized in Table 1. Quality of the PCR products was monitored by post-PCR melt-curve analysis. The expression of target genes was quantified using the −ΔΔ*C*t (Δ*C*t = Δ*C*t_target gene_−Δ*C*t_GAPDH_). Each experiment was repeated three times consecutively.

**Table 1.** Primer sequences for target genes.

Gene	Sense/Antisense	Sequences
TNF-α	Sense	5′-CACCACGCTCTTCTGTCTACTG-3′
	Antisense	5′-AGATAAGGTACAGCCCATCTGC-3′
IL-6	Sense	5′-CCCCAATTTCCAATGCTCTCC-3′
	Antisense	5′-CGCACTAGGTTTGCCGAGTA-3′
IL-1β	Sense	5′-GGGCCTCAAAGGAAAGAATC-3′
	Antisense	5′-TACCAGTTGGGGAACTCTGC-3′
IL-17A	Sense	5′-CTCCAGAAGGCCCTCAGACTAC-3′
	Antisense	5′-AGCTTTCCCTCCGCATTGACACAG-3′
IL-17F	Sense	5′-GAGGATAACACTGTGAGAGTTGAC-3′
	Antisense	5′-GAGTTCATGGTGCTGTCTTCC-3′
IFN-γ	Sense	5′-CTCAAGTGGCATAGATGTGGAAG-3′
	Antisense	5′-TGACCTCAAACTTGGCAATACTC-3′
GADPH	Sense	5′-CATGAGAAGTATGACAACAGCCT-3′
	Antisense	5′-AGTCCTTCCACGATACCAAAGT-3′

### Western blot

Total protein lysates were prepared from AR42J and IEC-6 cells and intestinal tissues from control, AP and OMT groups. Samples were loaded onto 10% SDS-polyacrylamide gels, transferred to polyvinylidene difluoride membranes (Millipore Corp., Bedford, MA, USA) and incubated with primary NF-κB (Cell Signaling Technology, Beverly, MA, USA), IκBα (Cell Signaling Technology), p-p38/p38 (Cell Signaling Technology), p-ERK/ERK (Cell Signaling Technology), ROR-γt (Santa Cruz, CA, UK), T-bet (Abcam, Cambridge, UK) and GAPDH (Proteintech, Chicago, IL, USA) antibodies overnight at 4 °C. Immunoreactive protein bands were visualized with an ECL detection kit (Millipore, Bedford, MA, USA). Each experiment was repeated three times.

### Immunohistochemistry

Immunohistochemistry (IHC) was performed as described previously (Zhang et al. 2012). Formalin-fixed, paraffin-embedded intestinal tissue sections were treated with 3% H_2_O_2_ for 20 min. Nonspecific antibody binding was then blocked using a specific blocking reagent for 30 min. CD44 (Abcam) and CD54 (Abcam) antibodies were incubated overnight at 4 °C. The corresponding secondary antibodies were incubated at room temperature for 20 min. Reaction products were visualized by incubation with 3,3′3-diaminobenzidine and then counterstained with haematoxylin. Staining intensity was scored as 0 (negative), 1 (weak), 2 (medium) and 3 (strong). Staining extent was scored as 0 (0%), 1 (1–25%), 2 (26–50%), 3 (51–75%) and 4 (76–100%) according to the percentage of the injury involved area as previously described (Sheng et al. 2017). The final IHC staining scores were determined by three professional pathologists.

### Statistics

Statistical analyses were performed using SPSS software 17.0 (SPSS, Chicago, IL, USA). The differences of MTT, qRT-PCR and western blot (WB) assays were expressed as mean ± SE and compared through Student’s *t*-test. Differences in gene expression in IHC assays were compared through paired sample non-parametric test. A value of *p* < 0.05 indicated statistical significance.

## Results

### OMT had no effect *in vitro* and *in vivo* without Arg treatment

**Figure 2.**
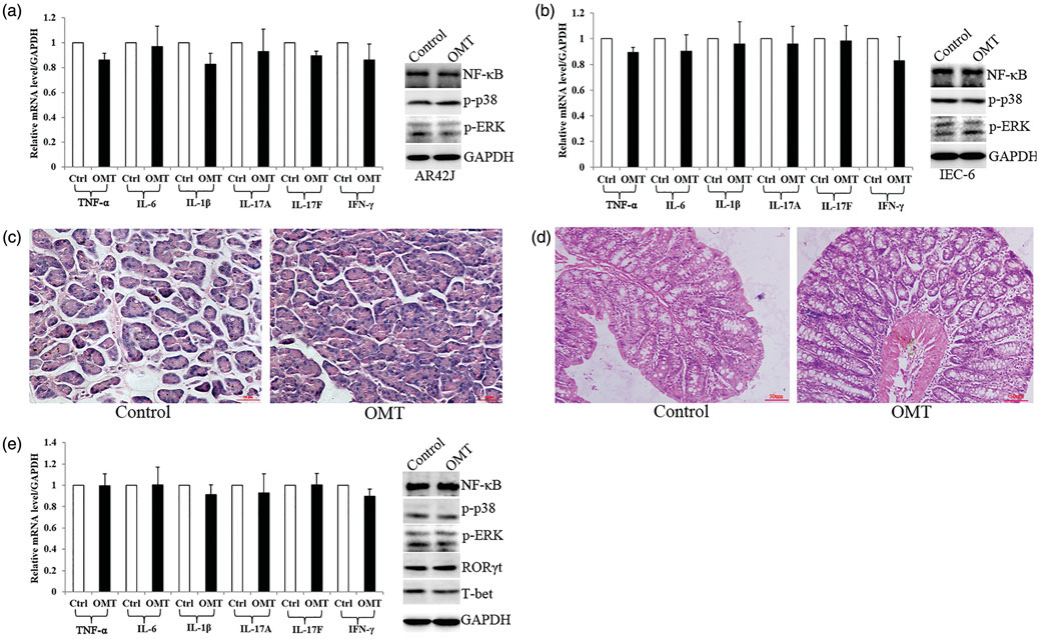
OMT effect *in vitro* and *in vivo* without Arg treatment. (a, b) OMT had no effect in regulating inflammation cytokines and MAPK signalling in both AR42J (a) and IEC-6 (b) cells. (c, d) OMT had no effect on the morphology of both pancreas (c) and intestine (d) (100×). (e) OMT had no effect in regulating inflammation cytokines and MAPK signalling *in vivo*. OMT: OMT treatment group; Ctrl: saline treatment group.

In normal rat pancreatic AR42J cells and intestinal IEC-6 cells, OMT (4 mg/mL) treatment had no effect in pro-inflammatory cytokines (TNF-α, IL-6 and IL-1β) mRNA expression compared with control group. IL-17A, IL-17F and IFN-γ, as the symbolic cytokines secreted by Th1 and Th17 cells, were unchanged as well (Figure 2(a,b)). Meanwhile, NF-κB, p-p38 and p-ERK protein expression showed no difference in the above two groups (Figure 2(a,b)).

*In vivo*, there was no difference in the morphology of both pancreas and intestine between OMT and control groups (Figure 2(c,d)). Moreover, TNF-α, IL-6, IL-1β, IL-17A, IL-17F, IFN-γ, NF-κB, p-ERK, p-p38 and transcription factors ROR-γt and T-bet released from Th1 and Th17 cells were also unchanged in both two groups (Figure 2(e)).

Taken together, OMT had no effect *in vitro* and *in vivo* without Arg which driven to promoted its potential role combining with Arg treatment.

### OMT inhibits Arg-induced inflammation and MAPK signalling *in vitro*

**Figure 3.**
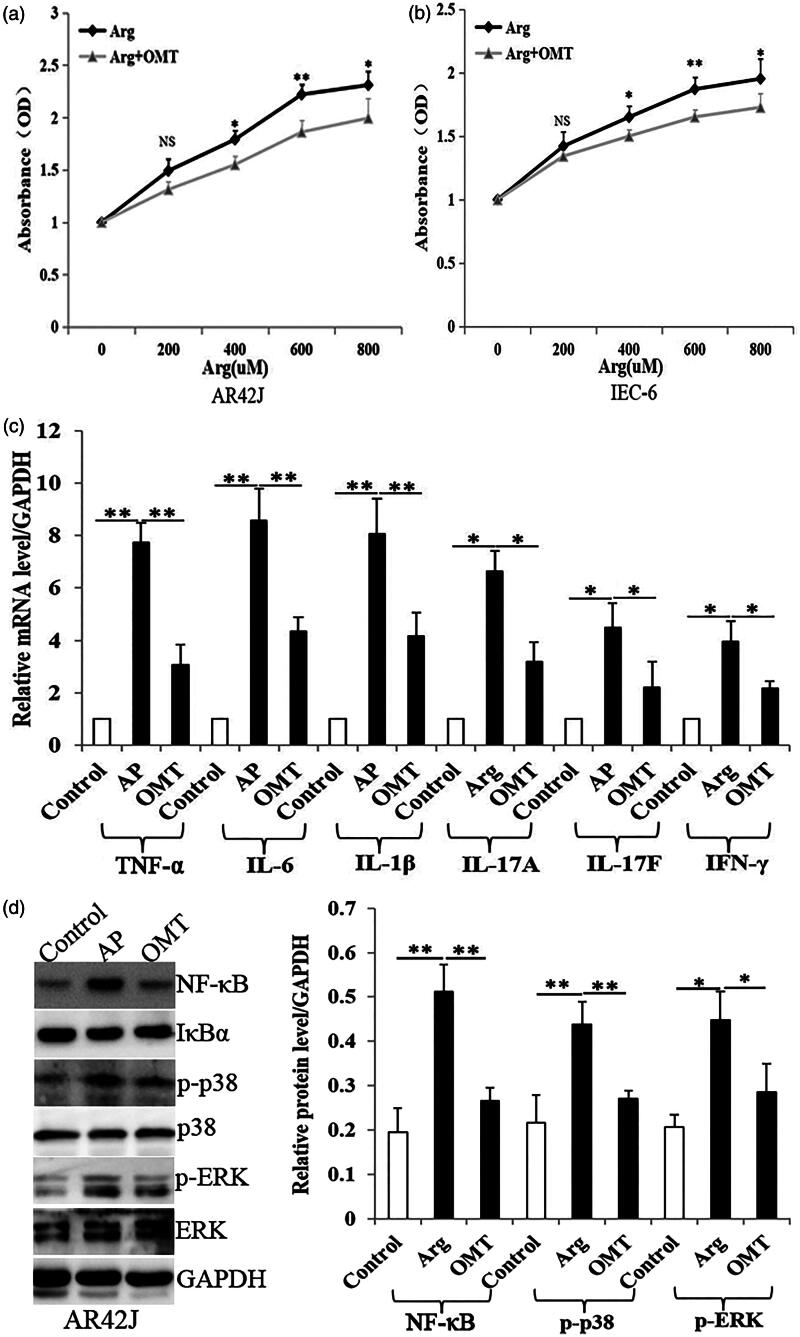
OMT inhibited Arg-induced inflammation and MAPK signalling *in vitro*. (a, b). Arg enhanced cell proliferation in a dose-dependent manner (from 400 to 800 µM) with MTT assays, which was inhibited by OMT treatment (4 mg/mL). (c) The mRNA levels of TNF-α, IL-6, IL-1β, IL-17A, IL-17F and IFN-γ in control, AP and OMT groups in pancreatic AR42J cells by qRT-PCR assays. (d) The protein levels of NF-κB, p-p38 and p-ERK in control, AP and OMT groups in AR42J cells with WB assays. Bars indicate ± S.E. *p < 0.05 compared with the control. **p < 0.01 compared with the control. Ctrl: saline treatment group; AP: Arg treatment group; OMT: Arg plus OMT treatment group.

MTT showed that Arg treatment (48 h) enhanced cell proliferation in a dose-dependent manner in both AR42J and IEC-6 cells (Figure 3(a,b)). The EC_50_ in Arg-treated AR42J and IEC-6 cells was 633.9 ± 31.4 µM and 571.3 ± 40.4 µM, respectively. However, OMT (4 mg/mL) significantly inhibited Arg-induced cell growth, especially at 600 µM of Arg treatment (*p* < 0.01) (Figure 3(a,b)). Based on the above results, we used the same situation for later PCR and WB assays *in vitro*.

**Figure 4.**
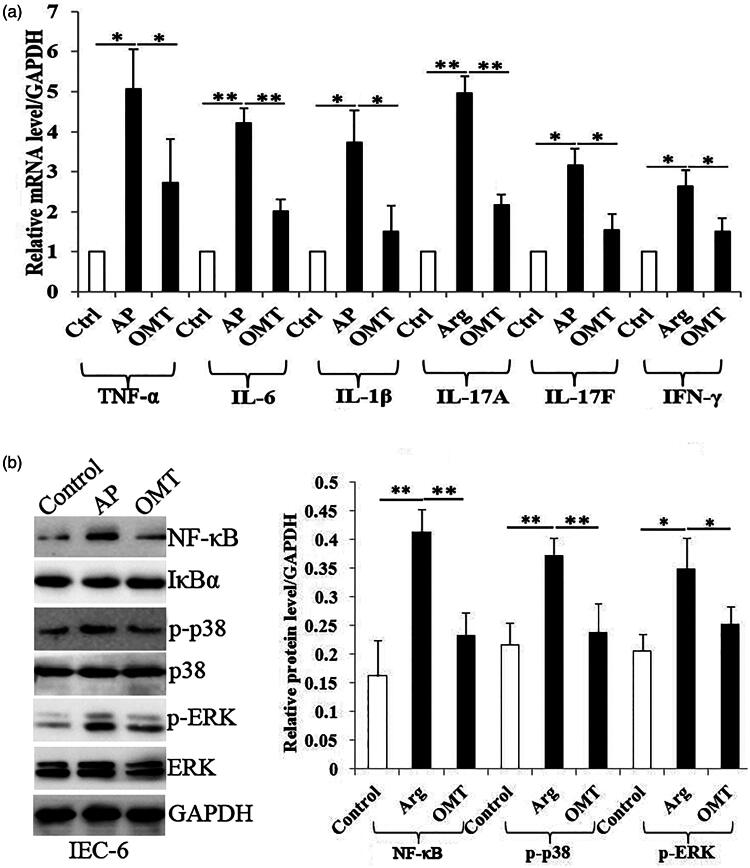
OMT inhibited Arg-induced inflammation and MAPK signalling *in vitro*. (a) The mRNA levels of TNF-α, IL-6, IL-1β, IL-17A, IL-17F and IFN-γ in control, AP and OMT groups in IEC-6 cells using qRT-PCR assays. (b) The protein levels of NF-κB, p-p38 and p-ERK in control, AP and OMT groups in IEC-6 cells using WB assays. Bars indicate ± S.E. *p < 0.05 compared with the control. **p < 0.01 compared with the control. Ctrl: saline treatment group; AP: Arg treatment group; OMT: Arg plus OMT treatment group.

In pancreatic AR42J cells, Arg (600 µM) significantly induced pro-inflammatory cytokines TNF-α, IL-6 and IL-1β mRNA expression. Meanwhile, IL-17A, IL-17F and IFN-γ were also upregulated by Arg (600 µM). However, OMT (4 mg/mL) significantly reversed the increase of above cytokines induced by Arg (Figure 3(c)), compared with AP group. Similar results were also observed in intestinal IEC-6 cells (Figure 4(a)).

WB showed that Arg (600 µM) significantly induced NF-κB, p-p38 and p-ERK protein expression. However, OMT (4 mg/mL) reversed Arg-induced increase of NF-κB, p-p38 and p-ERK protein levels (Figure 3(d)), compared with AP group. The same results were also repeated in IEC-6 cells (Figure 4(b)).

Taken together, OMT inhibits Arg-induced inflammation and MAPK/NF-κB signalling *in vitro*.

### Histopathology and morphology of the pancreas and the small intestine

**Figure 5.**
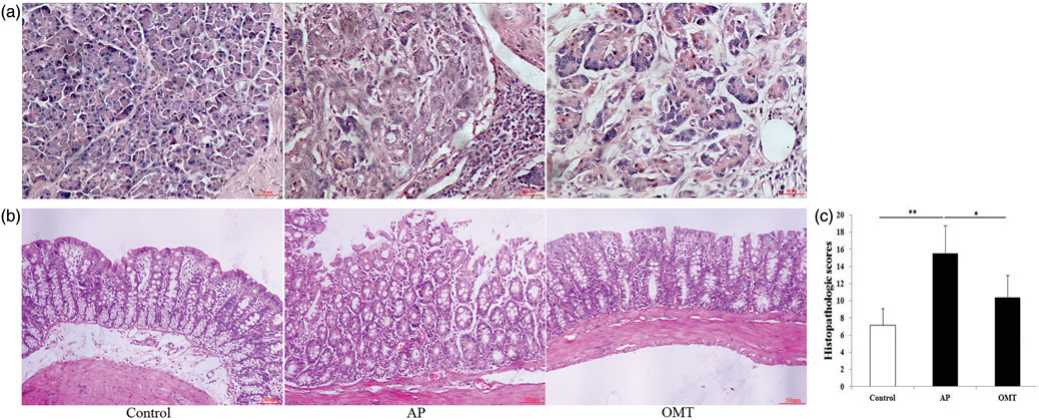
The inflammatory histologic changes of pancreas and intestine in control, AP and OMT groups, respectively. (a) Pancreatic histopathology in the in control, AP and OMT groups (100×). (b) Intestinal histopathology in the intestine of control, AP and OMT groups (100×). (c) Histopathologic scoring of intestinal injury was plotted in control, AP and OMT groups. Bars indicate ± S.E. *p < 0.05 compared with the control. **p < 0.01 compared with the control. Ctrl: saline treatment group; AP: Arg treatment group; OMT: Arg plus OMT treatment group.

Arg (250 mg/mL) successfully induced different severities of AP in 10 rats combined with the disruption of the intestinal barrier in histopathology and morphology assays (Figure 5).

The control group exhibited normal histological features of the pancreas (a normal architecture filled with acinar cells) (Figure 5(a)). The AP group revealed a tissue damage characterized by edema, inflammatory cell infiltrates and acinar cell necrosis (Figure 5(a)). However, AP with OMT (50 mg/kg) treatment resulted in a significant amelioration of pancreatic injury (low inflammatory cell infiltrates and relative complete acinus morphology) (Figure 5(a)).

Histology of intestinal tissue from the control group rats revealed normal mucosal architecture with similar histologic scores (Figure 5(b,c)). The AP group showed severe inflammatory injury to the intestinal mucosa with denuded villi, disintegrated lamina propria, exposed capillaries, and the infiltration of neutrophil and macrophage (Figure 5(b,c)). Histological examination of intestinal tissue in OMT group revealed only capillary congestion and mild epithelial lifting from the lamina propria, compared with AP group (Figure 5(b,c)).

Taken together, OMT attenuates Arg-induced pancreatic and intestinal histologic damage.

### OMT inhibits Arg-induced inflammatory *in vivo*

**Figure 6.**
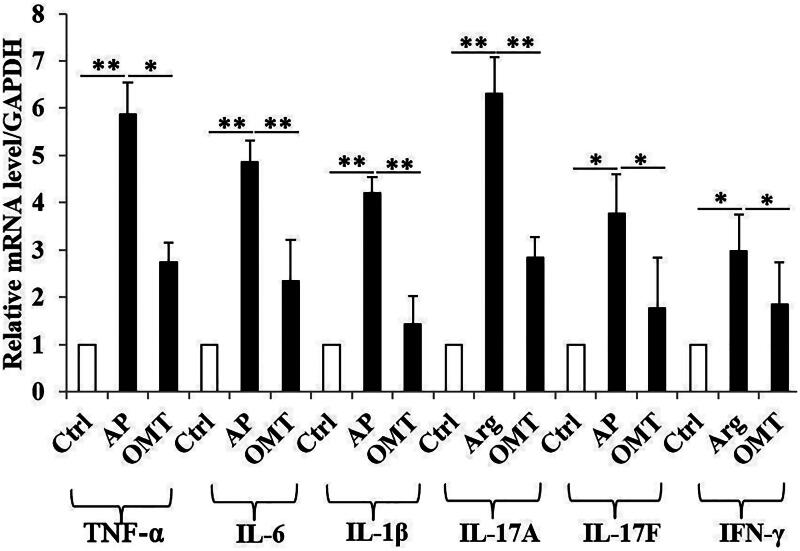
OMT inhibited Arg-induced inflammation *in vivo*. The mRNA levels of TNF-α, IL-6, IL-1β, IL-17A, IL-17F and IFN-γ in control, AP and OMT groups *in vivo* by qRT-PCR assays. Bars indicate ± S.E. *p < 0.05 compared with the control. **p < 0.01 compared with the control. Ctrl: saline treatment group; AP: Arg treatment group; OMT: Arg plus OMT treatment group.

Arg (250 mg/mL) group showed a significant increase of TNF-α, IL-6, IL-1β, IL-17A, IL-17F and IFN-γ mRNA levels in intestinal tissues, compared with control group. However, OMT (50 mg/kg) reversed the increase of above inflammatory cytokines induced by Arg, compared with AP group (Figure 6).

### OMT inhibits Arg-induced NF-κB, MAPK signalling and Th1/Th17-related transcription factor expression *in vivo*

**Figure 7.**
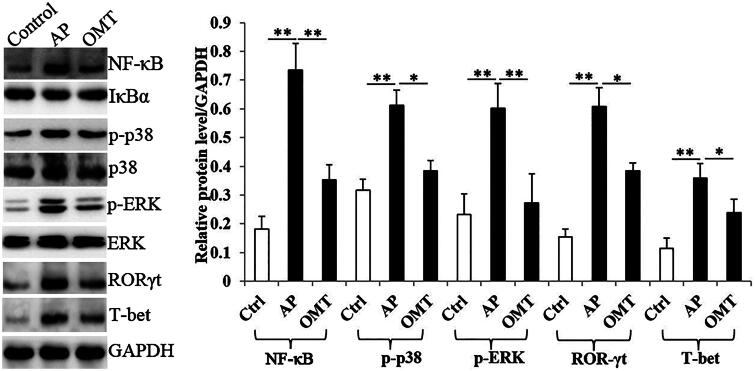
OMT inhibited Arg-induced NF-κB, MAPK signalling and Th1/Th17-related transcription factor expression *in vivo*. The protein levels of NF-κB, p-p38, p-ERK, ROR-γt and T-bet in control, AP and OMT groups *in vivo* using WB assays. Bars indicate ± S.E. *p < 0.05 compared with the control. **p < 0.01 compared with the control. Ctrl: saline treatment group; AP: Arg treatment group; OMT: Arg plus OMT treatment group.

The Arg (250 mg/mL) group showed a significantly increase of NF-κB, p-p38 and pERK protein levels in intestinal tissues compared with the control group. However, the increase of the above proteins induced by Arg in AP group was partially reversed by OMT (50 mg/kg) (Figure 7).

Since ROR-γt and T-bet are signature transcription factors and regulators in the differentiation and cytokines release of Th1 and Th17 cells, we further investigated OMT function in regulating ROR-γt and T-bet activity. WB showed that Arg significantly induced ROR-γt and T-bet protein expression, which were also reversed by OMT treatment (Figure 7).

### OMT inhibits Arg-induced CD44 and CD54-mediated inflammation *in vivo* by IHC

**Figure 8.**
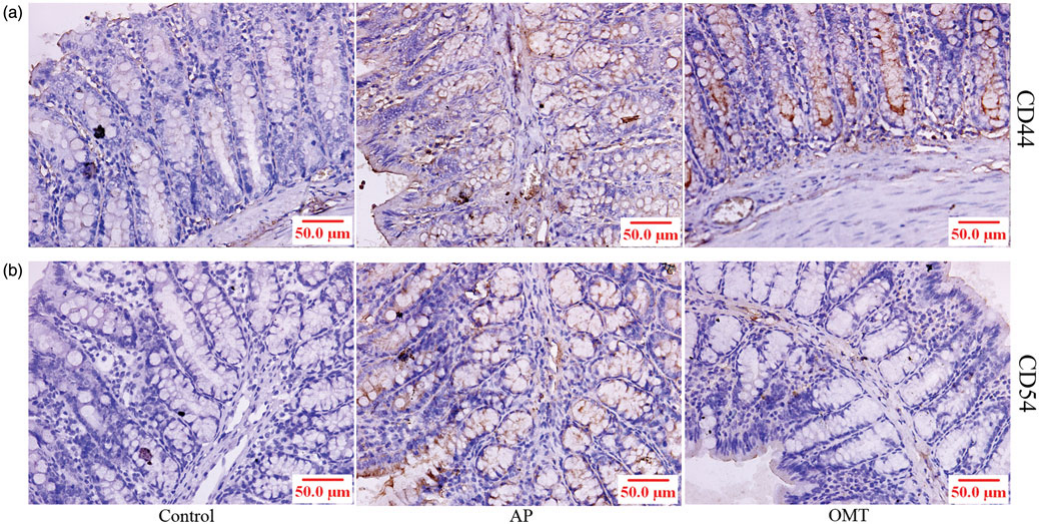
OMT inhibited Arg-induced CD44- and CD54-mediated inflammation *in vivo*. CD44 (a) and CD54 (b) expression in the intestine of control, AP and OMT groups using IHC assays (200×). Control: saline treatment group; AP: Arg treatment group; OMT: Arg combing with OMT treatment group.

IHC showed that both CD44 and CD54 were localized in the cytoplasm and membrane in intestinal tissues (Figure 8). A significant increase of CD44 and CD54 expression in intestinal tissues was shown in the Arg group (250 mg/mL) compared with the control group (*p* < 0.01; *p* < 0.01, respectively). However, OMT (50 mg/kg) partially inhibited Arg-induced CD44 and CD54 protein expression in the AP group (*p* < 0.05; *p* < 0.05, respectively).

## Discussion

OMT exhibits a protective effect in multiple agent-induced injuries in different animal models, including liver, heart, brain and intestine (Hong-Li et al. 2008; Liu et al. 2009; Guzman et al. 2013; Wen et al. 2014; Zhang et al. 2016a; Chen et al. 2017b). However, the molecular mechanism of OMT in Arg-induced AP and intestinal injury, to our knowledge, hasn’t been reported. In the current study, we first found that OMT inhibits Arg-induced AP and intestinal injury *in vitro* and *in vivo*.

Different chemical agents supply a pro- or anti-effect in Arg-induced AP in different strains of rat. For example, IFN-γ aggravated Arg-induced AP in Sprague-Dawley rats (Liu et al. 2017). Melatonin protects against Arg-induced AP in adult male Albino rats (Sadek and Khattab 2017). Inhibition of arginase activity ameliorated Arg-induced AP in Wistar rats (Biczó et al. 2010). Recent animal experiments and clinical studies showed the tight interaction between AP and intestinal barrier injury (Wang et al. 2009; Xu et al. 2014). Bacteria derived from intestinal injury in peripheral blood were closely linked to the severity of AP (Li et al. 2013). However, the effective treatment and potential molecular mechanism have not been fully elucidated. We first showed that OMT inhibited Arg-induced inflammation (TNF-α, IL-6, IL-1β and NF-κB), Th1/Th17 cells secreted cytokines (IL-17A/IL-17F and IFN-γ) and p38/ERK-MAPK signalling in both rat pancreatic AR42J and intestinal IEC-6 cells. In other studies, OMT sensitized HaCaT cells to the IFN-γ pathway and downregulated MDC, ICAM-1 and SOCS1 by activating p38, JNK and Akt (Gao et al. 2018). LPS induced NF-κB was significantly inhibited by OMT-pretreated MS1 cells (Lu et al. 2017). OMT inhibited the production of TNF-α, IL-1β and IL-6, suppressed the phosphorylation of IkBa in cytosol, decreased the nuclear levels of p65, and blocked ERK, p38 and JNK pathway in LPS-stimulated BV2 microglial cells (Dong et al. 2013).

Consistent with the results *in vitro*, OMT inhibited Arg-induced AP and intestinal injury *in vivo* along with the inhibition of Arg-induced inflammation (TNF-α, IL-6, IL-1β, NF-κB) and p38/ERK-MAPK signalling. OMT also protects other agent-induced intestinal injury. OMT protected against cirrhosis-associated intestinal mucosal damage via inhibiting NF-κB-mediated signalling and attenuated intestinal ischaemia/reperfusion injury in rats via inhibiting TNF-α and p-p38/MAPK signalling (Wen et al. 2014). Though the inhibition of OMT in ERK/MAPK signalling has not been reported in intestine, OMT inhibited TGFβ1-induced rat cardiac fibroblasts proliferation and myofibroblast transition through inhibiting p38/ERK-MAPK pathway (Zhao et al. 2008). NF-κB is the key regulator of inflammatory cytokines, including TNF-α, IL-1β and IL-6 (Santos et al. 2016). Meanwhile, a strong biological link between NF-κB and the MAPK pathway in inflammatory modulation is shown: galangin ameliorated cisplatin-induced nephrotoxicity by attenuating oxidative stress, inflammation and cell death in mice through inhibiting ERK and NF-κB signalling (Al-Hanbali et al. 2009). The anti-inflammatory effect of OMT was shown in LPS-induced BV2 microglia cells through inhibition of NF-κB and MAPK activation (Dong et al. 2013). Thus, OMT improved Arg-induced AP and intestinal injury involving NF-κB/MAPK-mediated inflammation.

In addition, this is the first study to show that that OMT inhibited Arg-induced IL-17A/IL-17F and IFN-γ cytokines and transcripts factors ROR-γt and T-bet expression. IFN-γ and IL17 are symbolic cytokines secreted by Th1 and Th17 cells, while T-bet and ROR-γt transcription factors are essential for Th1/Th17 differentiation and IFN-γ/IL17 activation (Chen et al. 2017b). OMT also protected against RA through inhibiting inflammation and regulating Treg/Th17 in CIA rats (Ma et al. 2017). Th1 and Th17 cell differentiation and function were restrained by OMT in DSS-induced colitis models (Chen et al. 2017b). The present results revealed that OMT executed an anti-inflammatory function in Arg-induced intestinal injury via inhibiting Th1/Th17-secreted cytokines.

Finally, OMT inhibited Arg-induced CD44 and CD54 (ICAM-1) expression *in vivo*. CD44 and CD54 play significant roles in inflammatory response *in vivo*. CD44 promoted inflammation and extracellular matrix production during arteriovenous fistula maturation (Kang et al. 2013). Specific inhibition of ICAM-1 effectively reduced bladder inflammation in rat with severe non-bacterial cystitis (Zhang et al. 2016b). An anti-human ICAM-1 antibody inhibited rhinovirus-induced exacerbations of lung inflammation (Traub et al. 2013). Meanwhile, intestinal reperfusion resulted in an increase of systemic ICAM-1 expression with marked organ variability in rats (Olanders et al. 2002). Therefore, OMT also inhibited Arg-induced intestinal injury via inhibiting CD44- and CD55-mediated inflammation.

**Figure 9.**
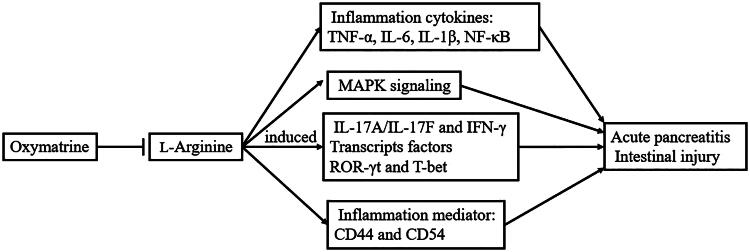
OMT inhibited Arg-induced AP involving intestinal injury *in vitro* and *in vivo* via regulating pro-inflammation cytokines and mediators, MAPK signalling, Th1/Th17 cytokines and corresponding transcript factors ROR-γt and T-bet expression.

As shown in Figure 9, we found that OMT inhibited Arg-induced AP and intestinal injury *in vitro* and *in vivo* via regulating pro-inflammation cytokines and mediators, MAPK signalling, Th1/Th17 cytokines and corresponding transcripts factors ROR-γt and T-bet expression. OMT is considered as a potential therapeutic agent in AP and intestine injury.
